# High frequency optical coherence tomography assessment of homogenous neck coverage by intrasaccular devices predicts successful aneurysm occlusion

**DOI:** 10.1136/neurintsurg-2019-014843

**Published:** 2019-04-29

**Authors:** Robert M King, Miklos Marosfoi, Jildaz Caroff, Giovanni J Ughi, Dale M Groth, Matthew J Gounis, Ajit S Puri

**Affiliations:** 1 New England Center for Stroke Research, Department of Radiology, University of Massachusetts Medical School, Worcester, Massachusetts, USA; 2 Department of Biomedical Engineering, Worcester Polytechnic Institute, Worcester, MA, USA; 3 Department of Interventional Neuroradiology, NEURI Center, Bicêtre Hospital, Le Kremlin-Bicêtre, France

## Abstract

**Background:**

High frequency optical coherence tomography (HF-OCT) is a novel intravascular imaging technology developed for use in the cerebral vasculature. We hypothesize that HF-OCT characterization of intrasaccular device neck coverage can prognosticate exclusion of the aneurysm from the circulation.

**Methods:**

Bifurcation and sidewall aneurysms were made in six dogs. Seven aneurysms were treated with next generation intrasaccular devices (NGID) and four with traditional platinum coils. HF-OCT was performed to interrogate gaps in the neck coverage, coil herniation, or acute thrombus formation. Animals were re-imaged at 7, 30, 90, and 180 days following aneurysm embolization. An automated image processing method segmented the devices at the neck of the aneurysm and quantified neck coverage. The largest coverage gap was used to predict aneurysm occlusion at 180 days.

**Results:**

No difference was found in occlusion rates between the coil and NGID groups (P=0.45). Successful segmentation of the NGID construct was achieved in all cases. A coverage gap >1 mm^2^ was found to predict failed aneurysm occlusion (P=0.047). This threshold was able to predict all cases of failed occlusion. The average number of devices needed to treat the aneurysm was lower in the NGID group (1.9 vs 6.75, P=0.009). HF-OCT showed strong agreement with scanning electron microscopy (bias 0.0024 mm^2^ (95% CI −0.0279, 0.0327)).

**Conclusions:**

HF-OCT enables precise and accurate measurement of coverage gaps at the neck of aneurysms treated with intrasaccular devices in vivo. We provide in vivo evidence that uniform aneurysm neck coverage by intrasaccular devices is critical for aneurysm occlusion.

## INTRODUCTION

Coil embolization of brain aneurysms is a widely established technique with level 1 evidence for ruptured aneurysms.^1^ However, one of the major concerns of coil embolization is recanalization due to incomplete healing, coil compaction, or aneurysm regrowth, warranting long term follow-up imaging.^2,3^ This is especially applicable for large and giant aneurysms,^4^ particularly those that are partially thrombosed.^5^ Clinical data have shown that approximately 20% of embolized aneurysms will recur, with up to 10% of all coiled aneurysms needing retreatment.^6^ Higher rates of recanalization are found in wide neck aneurysms due to poor neck coverage.^4^ Although this issue is now often addressed by flow diversion or stent assisted coiling (SAC),^7–9^ these techniques can incur additional risks of thromboembolic complications or complications associated with administration of dual antiplatelet therapy.^10^ Next generation coil^11^ or intrasaccular flow disruption^12^ designs have the potential to improve neck coverage and reduce the need for SAC.

Newly developed intrasaccular devices may improve neck coverage and following positive trials have received Food and Drug Administration approval. Preclinical studies in vitro showed that the reconstruction of the aneurysm neck is a critical component for achieving intra-aneurysmal flow reduction.^13^ High frequency optical coherence tomography (HF-OCT), a catheter based imaging technique specifically designed for use in tortuous neurovasculature, was recently introduced.^14^ Using near infrared light, HF-OCT acquires three-dimensional information from cerebrovascular arteries, providing micron level details of the arterial lumen morphology, intraluminal objects (eg, neurovascular devices and presence of thrombus), and the vessel wall microstructure. With a resolution approaching 10 μm, HF-OCT images can characterize in extraordinary detail the device and its relationship to the aneurysm neck at the time of implantation.

In this study, we hypothesized that gaps in the neck coverage visualized by HF-OCT at the time of implantation, but not visible by traditional X-ray imaging modalities (ie, DSA and CT), are predictive of failure of aneurysm occlusion. This would inform the need for modification of the treatment to ensure eventual aneurysm exclusion.

## METHODS

### Experimental procedures

All animal research procedures were approved by our university’s institutional animal care and use committee. Six purpose bred hound cross dogs (sex male, weight 20–25 kg) were used in this study. On the day of surgery, the animals were sedated and pretreated with intramuscular injections of a single dose of acepromazine (0.06 mg/kg), buprenorphine (0.02 mg/kg), and glycopyrrolate (0.01 mg/kg). Anesthesia was induced with an intravenous dose of propofol (3–4 mg/kg), and the animal was intubated. Anesthesia was maintained by mechanical ventilation of 1–3% isoflurane in oxygen. Additionally, ECG, SpO_2_, invasive or non-invasive arterial blood pressure, rectal temperature, and end tidal CO_2_ were continuously monitored and recorded every 15 min. Fentanyl patches (75 μg/hour) were used to manage postoperative pain following the aneurysm creation procedure. For subsequent interventional procedures, analgesia was provided with local administration of bupivacaine. Both sidewall and bifurcation anatomical variants were surgically created from a venous pouch, based on a previously described model.^15,16^ In brief, the midsection of the left common carotid artery was ligated, transected, and tunneled beneath the trachea where it was anastomosed end-to-side with an arteriotomy to the midsection of the right common carotid artery. A segment of the external jugular vein was anastomosed to the junction of the two arteries. The lateral wall aneurysm was constructed approximately 10 cm cranial to the newly constructed bifurcation aneurysm on the right common carotid artery. A total of six sidewall and six bifurcation aneurysms were created.

Aneurysm treatment was initiated at a minimum of 21 days post aneurysm creation. A 2:1 randomization was used to determine device selection: either embolization with a next generation intrasaccular device (NGID) or traditional coil embolization (Axium; Medtronic Neurovascular, Irvine California, USA). For traditional coil embolization, aneurysms were packed to achieve complete obliteration of the aneurysm with the goal of preventing herniation. We did not permit assist devices (balloon remodeling or stents) to reduce confounding variables, as well as determine the ability of the NGID to reconstruct the aneurysm neck. For NGID embolization, one or two devices were inserted at the discretion of the neurointerventional surgeon. Once the aneurysm was considered successfully packed, a prototype HF-OCT imaging device (Vis-M™; Gentuity LLC, Sudbury Massachusetts, USA) compatible for delivery through a 0.017 inch microcatheter, was navigated distal to the aneurysm. A 6 F balloon catheter was then inflated in the proximal right common carotid artery and the HF-OCT pullback was performed concurrently with contrast injection (5 mL/s for 20 mL), clearing the artery of residual blood. A balloon catheter was used to arrest very high afferent flow through the remaining carotid artery for approximately 5 s. HF-OCT images were generated real time, and displayed live during image acquisition. After both aneurysms were treated and imaged, DSA and high resolution cone beam CT (VasoCT)^17^ data were acquired for each aneurysm. DSA, HF-OCT, and VasoCT imaging was repeated at 7, 30, 90, and 180 days following implant to interrogate progressive healing of the aneurysm and capture conformational changes of the devices.

At day 180, the animals were euthanized and perfused with saline followed by 4% paraformaldehyde. After overnight immersion fixation in 2.5% glutaraldehyde, the explants were rinsed in 0.1 M cacodylate buffer. The arteries were longitudinally cut under a dissecting microscope to expose the neck of the aneurysm, and then dehydrated through a graded series of ethanol concentrations (up to 100%) followed by critical point drying in carbon dioxide. The arterial samples were mounted on to aluminum stubs, grounded with silver conductive paste, sputter coated with gold/palladium, and imaged with scanning electron microscopy (SEM) (Quanta 200 MKII FEG; FEI, Hillsboro, Oregon, USA).

### Image analysis

**Figure 1:**
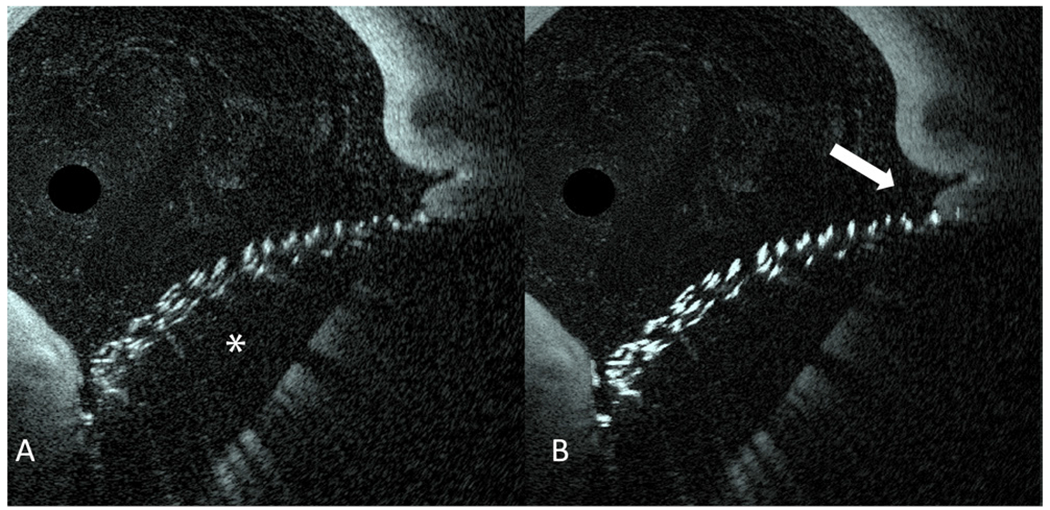
High frequency optical coherence tomography (HF-OCT) cross sectional images before (A) and after (B) the segmentation procedure, illustrating an accurate classification of the implant: (*) denotes the aneurysm. HF-OCT shows a small gap on the side of the neck (arrow), filled with uncleared blood.

For quantification of HF-OCT datasets, an inhouse developed automated segmentation algorithm was applied to classify image pixels into three categories: lumen pixels, clot or tissue, and device. First, the neck of the aneurysm was found by manually tracing the vessel wall and identifying the neck. Once the neck was located, the images were remapped into polar coordinates to proceed with the classification. A multi-parametric classification method based on the local gradient, threshold signal value, shadows trailed by objects that completely block the HF-OCT emitted light, and the cumulative sum of the pixel value from the center point was applied. An example of the classification results is shown in figure 1. Once the classification was complete, the results were reviewed and approved by an expert image reader. All gaps in the coverage of the entire neck of the aneurysm were identified, and from this, the largest gap was measured. Being a high resolution imaging modality (ie, with a spatial resolution approaching 10 μm),^14^ HF-OCT could depict and quantify the presence of both large and minor gaps, that cannot be visualized by DSA or VasoCT.

The primary outcome of the study was aneurysm occlusion determined using DSA after 180 days. The occlusion score was dichotomized to a good outcome (fully occluded) or bad outcome (any residual filling) and compared with Fisher’s exact t test. The number of devices implanted was compared with a non-parametric Mann–Whitney U test due to the non-normal distribution of the number of coils used. A threshold analysis of HF-OCT prediction of occlusion was applied and Fisher’s exact t test was used to determine if HF-OCT predicted occlusion of the aneurysm assessed with DSA. Finally, a Bland-Altman graph was generated to look at the bias and limits of agreement between HF-OCT and SEM. All statistical calculations were done in R 3.5.1 (the R Foundation).

## RESULTS

All aneurysms were successfully treated (n=11) except for one sidewall aneurysm (n=1) that was partially thrombosed on the day of surgery and therefore excluded from the study. Baseline aneurysm characteristics between the aneurysms treated with NGID and coils were found to be not significantly different (table 1). Aneurysms treated with coils trended towards a slightly higher occlusion score immediately following implant although it was not significantly different (P=0.09) (table 2). The number of devices deployed was greater for the aneurysms coiled than those treated with NGID (6.75 vs 1.9, respectively; P=0.009, Mann–Whitney U test). The NGID formed a stable construct at the level of the neck with no case of herniation into the parent artery whereas there was evidence of coil herniation in 75% of aneurysms (P=0.024). On final follow-up 180 days after treatment, DSA occlusion scores were not statistically different between coils and the NGID device (P=0.45, Fisher’s exact t test).

**Figure 2:**
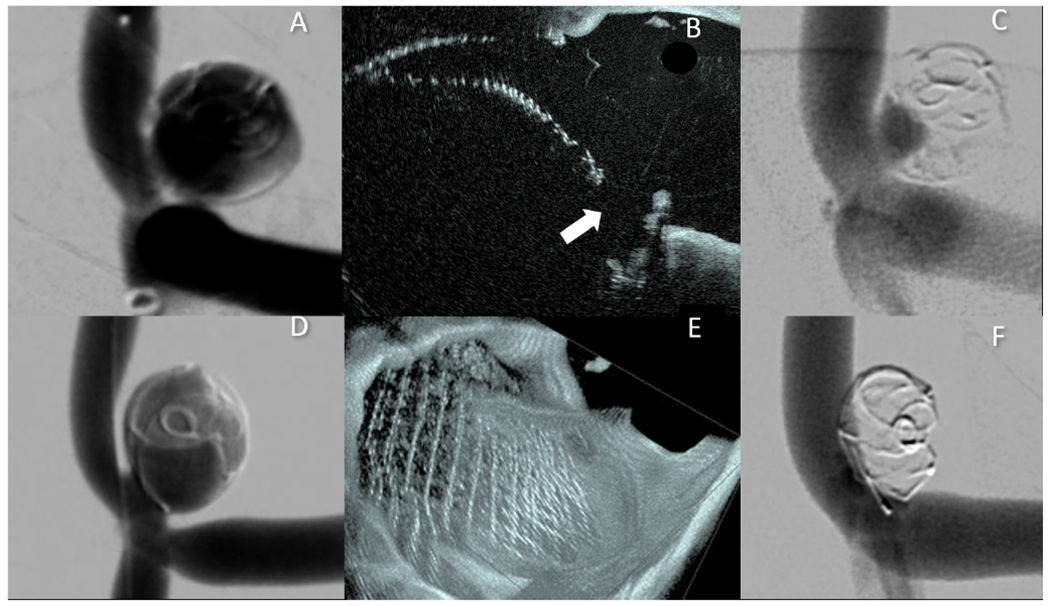
Control angiography of a bifurcation aneurysm after next generation intrasaccular device (NGID) embolization (A). High frequency optical coherence tomography (HF-OCT) shows a gap in the neck coverage (B, arrow), leading to an aneurysm neck remnant at day 180 (C) and compaction of the device. Angiography of a second bifurcation aneurysm embolized with a NGID (D). Three-dimensional HF-OCT data show evidence of complete and uniform neck coverage (E). The aneurysm was fully occluded after 30 days and remained stable throughout the observation period of 180 days (F).

**Figure 3:**
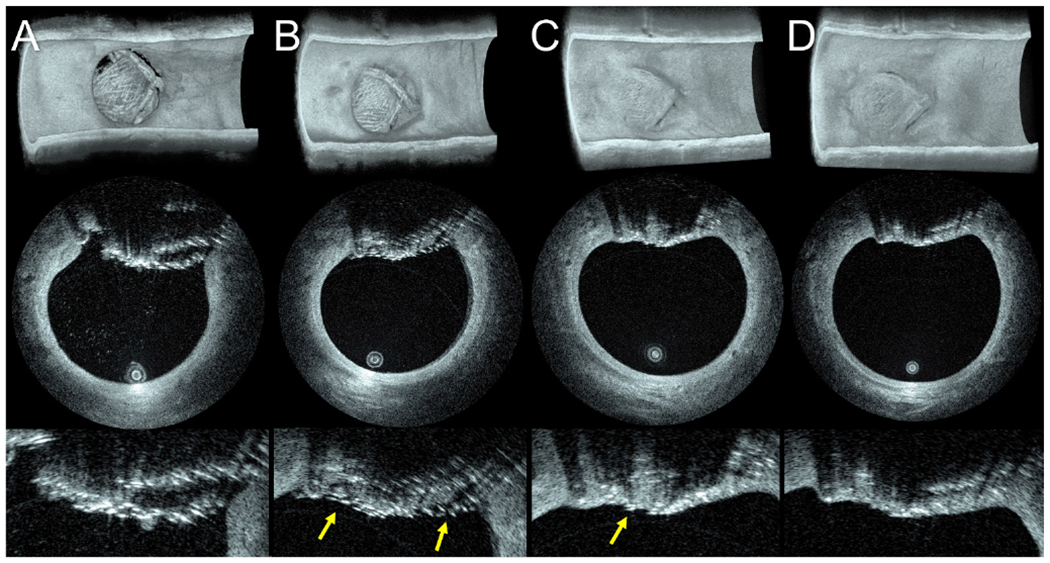
High frequency optical coherence tomography (HF-OCT) imaging of progressive in vivo aneurysm neck remodeling: baseline versus 7, 30, and 180 days following implantation. Three-dimensional HF-OCT data acquired at baseline show complete coverage of the aneurysm neck (A). Follow-up at 7 days shows healing progression with a significant amount of uncovered struts exposed to the blood flow (B, arrows). HF-OCT imaging at 30 days shows tissue growth over the device (C); the arrow points to a small area of an uncovered metallic strut. HF-OCT imaging at 180 day shows additional healing progression and no neck remnants (D).

HF-OCT findings showed that all gaps of neck coverage larger than 1 mm^2^ at the time of the implant were predictive of persistent aneurysm dome filling culminating in device compaction at 6 months (P = 0.047, Fisher’s exact t test) (table 2). A representative set of treated bifurcation aneurysms is shown in figure 2. All aneurysms with a large coverage gap failed to occlude (contrast penetration into the aneurysm dome) at 6 months whereas uniform coverage resulted in complete occlusion within 30 days that remained stable throughout the duration of surveillance (figure 3).

**Figure 4:**
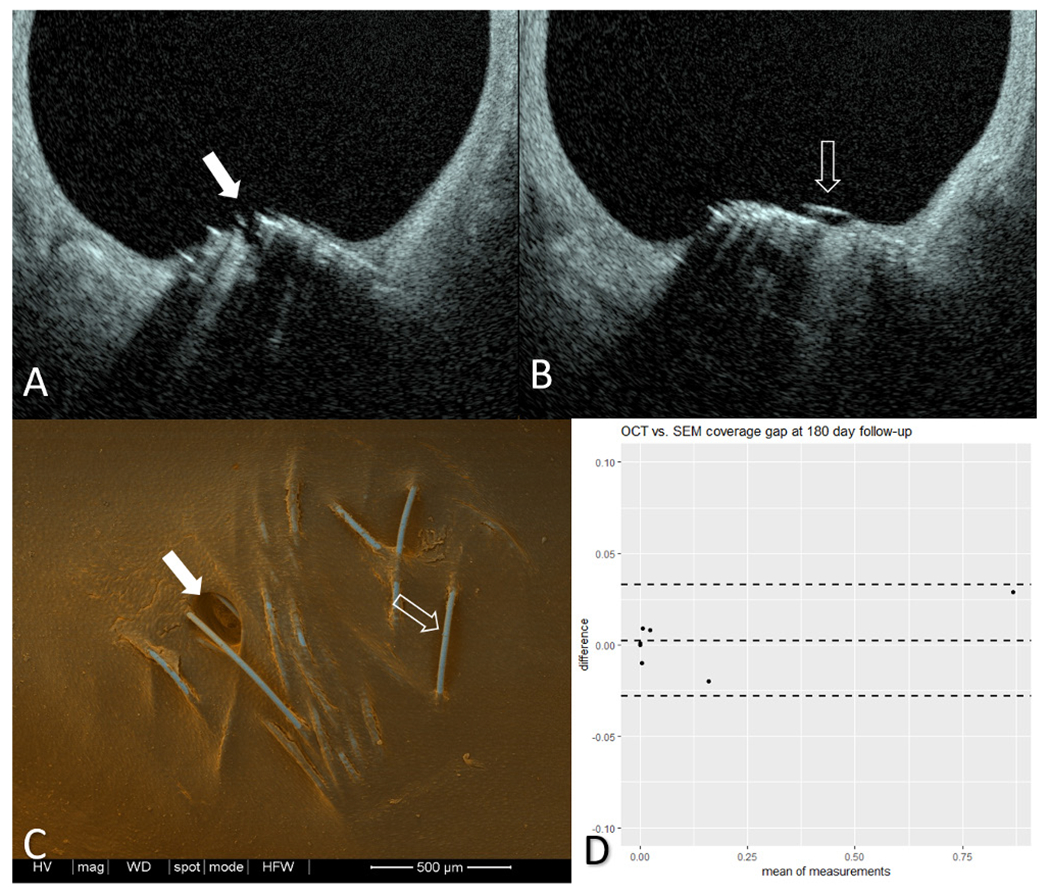
High frequency optical coherence tomography (HF-OCT) (A, B) and scanning electron microscopy (SEM) (C) images of the next generation intrasaccular device, 180 days following implant. Images show a small area of incomplete tissue coverage (A, solid arrows) and an uncovered metallic strut (B, open arrow). En face SEM imaging of the device (C); open arrow, uncovered strut, and solid arrow, gap in tissue coverage, confirms these findings. Bland–Altman plot of HF-OCT versus SEM for the measurement of the coverage gap at 180 days.

**Figure 5:**
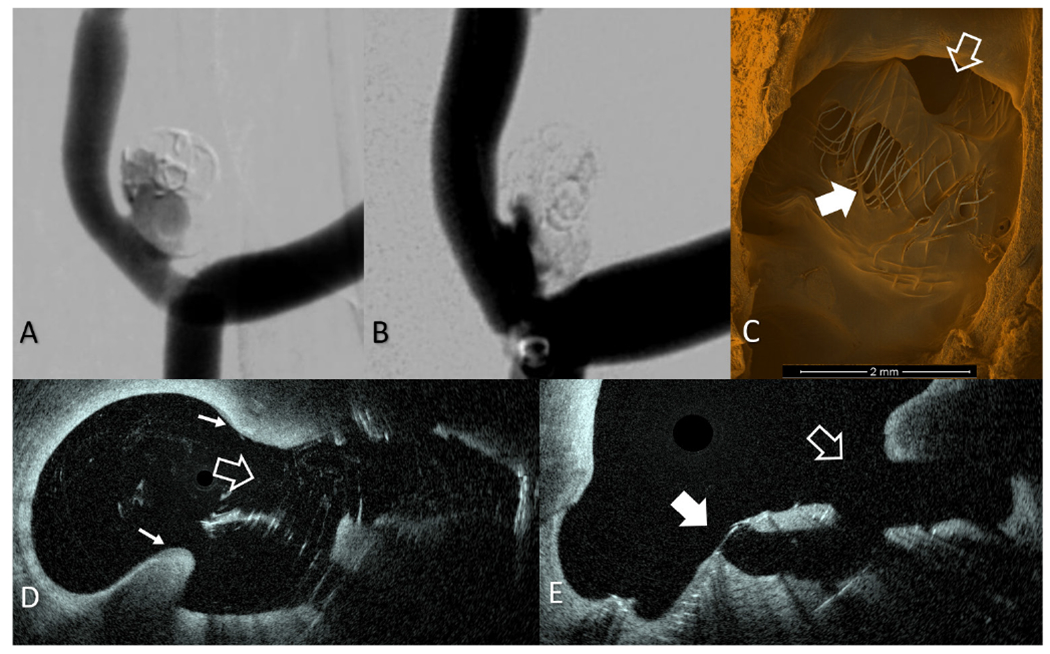
Control angiography following implant (A) showing contrast entering the dome of the aneurysm treated with a next generation intrasaccular device. Follow-up angiogram at 180 days demonstrates residual filling of the aneurysm dome and compaction of the device (B). Scanning electron microscopy (SEM) reveals a coverage gap at the neck (C, open arrow), and uncovered struts (solid arrow). Day 0 high frequency optical coherence tomography (HF-OCT) (D) shows a large coverage gap (D , open arrow) at the aneurysm neck (defined by the small, solid arrows). HF-OCT at day 180 clearly depicts the neck coverage gap (E, open arrow) and the uncovered struts (E, solid arrow) seen on the post mortem SEM.

SEM and HF-OCT findings at 180 days were qualitatively and quantitatively compared. Measurement of the coverage gaps in the NGID construct were obtained for both modalities. HF-OCT was found to have a very high level of agreement with SEM (figure 4), with no bias, small limits of agreement (bias 0.0024mm^2^; 95% CI −0.0279, 0.0327), and no significant difference. Qualitative analysis showed that when small areas of incomplete endothelization of the NGID device on SEM were identified, and the corresponding slices from the HF-OCT were examined, the images showed similar findings (figures 4 and 5).

## DISCUSSION

Coiling of intracranial aneurysms has been shown to be a safe and effective treatment. Despite an excellent safety profile, there is a known risk of recurrence and potential need of retreatment. The balloon remodeling technique remains an important option for coiling wide neck aneurysms.^18,19^ To reduce the chances of aneurysm recanalization, SAC has often been adopted. However, SAC techniques may lead to an additional chance of thrombotic complications, as high as 16%.^20^ The development of new intrasaccular devices offers potential for safer aneurysm treatment that avoids the use of dual antiplatelet therapy, uses fewer devices, and may reduce procedure time. As an additional benefit, intrasaccular devices often require fewer implants than traditional aneurysm coils (1.9 vs 6.75 in this study). Preliminary experience with these devices has shown promising results^21^ but also concerns regarding recurrence, particularly when only one device is used.^22^ The specific mechanism for intrasaccular device failure to achieve occlusion may be related to lack of mechanical support from tight packing to resist transient hemodynamic forces leading to fatigue, failure to reconstruct the neck, or related to patient specific factors. In a controlled experiment, we show that small gaps in neck coverage delay aneurysm healing and lead to device compaction.

Following coil treatment, current predictive tools of aneurysm occlusion include packing density, aneurysm volume, and Raymond–Roy classification,^23,24^ with post-treatment angiographic classification showing the greatest OR. Due to photon starvation in a densely packed aneurysm, contrast penetration into the aneurysm neck and dome may not be fully appreciated, a limit of current imaging capabilities. Furthermore, given the reduced X-ray attenuation of these newer intrasaccular devices, a new classification paradigm may be required for outcome prediction. In this study, we have shown that the measurement of the largest gap in the neck coverage, obtained using high resolution intravascular imaging, predicts the efficacy of aneurysm embolization. Altogether, the results of this study illustrated the potential benefits of performing HF-OCT acquisition prior to device detachment to further inform treatment decisions. Using in vitro vascular models, it was shown that effective neck coverage of intrasaccular devices is critical for hemodynamic modification,^13,25^ a finding consistent with the in vivo results presented herein. As such, the use of HF-OCT imaging combined with the ability to modify device placement can provide procedural guidance for an optimized deployment, significantly increasing the chances of obtaining complete aneurysm occlusion.

Numerical simulations have also been proposed to predict recurrence following coil embolization and applied successfully to clinical cases.^26^ These approaches rely on important procedural information as well as certain assumptions about the implant (such as uniform distribution or resistance to flow). Herein, we demonstrate that HF-OCT can provide imaging data for subsequent quantitation of spatial variations in aneurysm neck coverage. These data may well complement numerical strategies by providing essential information for accurate modeling.

This study was limited by a relatively small sample size. Despite the low number of NGID devices, a threshold of 1 mm^2^ gap correctly classified the two cases in which the treatment failed. Furthermore, the remaining five cases with successful occlusion did have gaps in neck coverage but not exceeding this threshold and were therefore also correctly classified. Using a larger sample size will enable a more sensitive threshold to be established. Traditional coils often herniated from these wide neck aneurysms inhibiting a full three-dimensional reconstruction of the coil structure at the level of the neck due to imaging catheter positioning in the vessel lumen and optical attenuation by the coils. Image quality coupled with a limited sample size did not allow a meaningful analysis of traditional coil embolization in all cases.

## CONCLUSIONS

With the advent of HF-OCT, an informed and accurate prediction of treatment efficacy may be possible. In this study, HF-OCT enabled a quantitative analysis of the aneurysm neck for a new class of intrasaccular devices that is predictive of aneurysm occlusion. We showed that the presence of gaps with an area larger than 1 mm^2^ leads to persistent aneurysm filling and that homogenous neck reconstruction with no gaps larger than this threshold reliably predicts aneurysms occlusion. The results of this study demonstrate the feasibility of high resolution intravascular imaging for the assessment of acute device implantation, procedural guidance, and ability to prognosticate complete aneurysm occlusion.

## Figures and Tables

**Table 1:** Baseline aneurysm characteristics and device implant location

Animal No	Sidewall				Bifurcation			
	Dome diameter (mm)	Height diameter (mm)	Neck width (mm)	Device	Dome diameter (mm)	Height diameter (mm)	Neck width (mm)	Device
Animal No 1	4.58	4.55	<1	Not treated	11.14	11.09	5.12	Coils

Animal No 2	9.22	6.67	2.88	Coils	8.75	8.21	3.60	NGID

Animal No 3	7.11	6.24	3.28	Coils	8.01	7.99	3.60	NGID

Animal No 4	8.80	7.24	5.12	NGID	9.78	6.96	3.90	NGID

Animal No 5	5.27	3.52	3.44	NGID	8.13	8.91	4.24	Coils

Animal No 6	5.07	3.20	4.00	NGID	9.92	6.96	4.10	NGID

NGID, next generation intrasaccular device.

**Table 2:** Largest coverage gap in the next generation intrasaccular device construct as it compares wth the implant, and final DSA occlusion scores^3^

Animal No	Location	Device	No of implants	Herniation (Y/N)	Largest coverage gap at implant (mm^2^)	Implant DSA score^3^	180 day DSA outcome score^3^
Animal No 1	Bifurcation	Coils	11	Y	N/A	2	2

Animal No 2	Sidewall	Coils	6	N	N/A	2	1
	Bifurcation	NGID	2	N	1.57	3	3

Animal No 3	Sidewall	Coils	4	Y	N/A	2	1
	Bifurcation	NGID	2	N	0.18	3	1

Animal No 4	Sidewall	NGID	1	N	0.55	2	1
	Bifurcation	NGID	2	N	1.30	3	3

Animal No 5	Sidewall	NGID	3	N	0.09	3	1
	Bifurcation	Coils	6	Y	N/A	3	1

Animal No 6	Sidewall	NGID	2	N	0.08	3	1
	Bifurcation	NGID	1	N	0.12	3	1

Due to artifacts in imaging, it was not possible to perform this analysis on the standard coils.

NGID, next generation intrasaccular device.
